# Next Generation Sequencing for the Differentiation of Benign and Malignant Biliary Strictures

**DOI:** 10.1111/liv.70865

**Published:** 2026-09-05

**Authors:** Antonia Gillmeister, Julia Husman, Renate Schmelz, Stefan Brückner, Jochen Hampe, Daniela Aust, Sebastian Zeissig

**Affiliations:** ^1^ Department of Medicine I, University Hospital Carl Gustav Carus Dresden Technische Universität Dresden (TUD) Dresden Germany; ^2^ Institute for Pathology, University Hospital Carl Gustav Carus Dresden Technische Universität Dresden (TUD) Dresden Germany; ^3^ Department of Visceral Surgery and Medicine, Inselspital – Bern University Hospital University of Bern Bern Switzerland

**Keywords:** biliary specimens, biliary strictures, BiliSeq, ERCP, next generation sequencing (NGS)

## Abstract

The differentiation between benign and malignant biliary strictures remains a significant clinical challenge. Recent studies have suggested that next generation sequencing (NGS) can improve the diagnostic accuracy. However, evidence on its performance in routine clinical practice is limited. Therefore, validation of the diagnostic value of NGS using real‐world clinical data is warranted. We compared the performance of NGS analysis of cholangiocellular carcinoma (CCC)‐associated mutations in endoscopic biopsies with that of histopathology, CA19‐9 and cross‐sectional imaging. NGS showed higher sensitivity for malignancy (65%) compared to histopathology (52%) at similar specificity (96% vs. 100%). The combination of NGS and histopathology further increased sensitivity for malignancy to 78% (*p* = 0.03) at similar specificity (96%). Our data provide support for the use of NGS in the diagnostic workup of biliary strictures.

## Key Points

- NGS analysis of biliary biopsies improves the sensitivity for detecting malignancy in indeterminate biliary strictures compared with histopathology alone, without compromising specificity.
- Combining NGS with histopathological analysis further increases diagnostic sensitivity while maintaining high specificity.
- Despite the limitations of a small cohort and limited follow‐up, our findings support the integration of NGS into the diagnostic workup of biliary strictures of unknown aetiology.

## Lay Summary

The early differentiation between benign and malignant biliary strictures remains a significant clinical challenge despite recent improvements in imaging and pathological evaluation. Detecting cholangiocellular carcinoma‐associated mutations in endoscopic biopsies can improve diagnostic sensitivity beyond conventional histopathology. Therefore, our study provides real‐world validation of using next generation sequencing as a diagnostic tool and confirms the higher sensitivity of NGS and the further improvement when both methods were combined. Integrating NGS into the diagnostic workup of biliary strictures could especially enhance early cancer detection. However, the limited sample size of this study may restrict the generalizability of the results, and larger prospective studies are needed for further validation.

**Abbreviations**
CCC: Cholangiocellular Carcinoma
ERC: Endoscopic Retrograde Cholangiography
LOD: Limit of detection
NGS: Next Generation Sequencing
PSC: Primary Sclerosing Cholangitis
VAF: Variant Allele Frequency
VUS: Variants of unknown significance

## 1 Introduction

The differentiation of benign and malignant biliary strictures remains a challenge in clinical diagnostics [1], but is of paramount importance due to the highly aggressive nature of CCC and the need for early disease detection and treatment [2]. Cross‐sectional imaging, histopathological evaluation, and the assessment of tumour markers (CA19‐9), either alone or in combination, are of limited sensitivity for detection of malignancy, particularly in the context of primary sclerosing cholangitis (PSC) [3]. Furthermore, cross‐sectional imaging and CA19‐9 are also of limited specificity in distinguishing benign from malignant strictures [3]. Since CCC arises from somatic mutations, recent work investigated whether NGS of biliary biopsies [4] or cell‐free DNA [5], followed by screening for mutations in genes recurrently affected in CCC can improve the sensitivity for detection of malignancy. Indeed, this work revealed that NGS analysis of cancer‐associated genes in biliary biopsies improves the sensitivity for the detection of malignant strictures compared to histopathological evaluation alone (73% vs. 48%), while it does not compromise specificity (100% for NGS, 99% for histopathology) [4]. The combination of NGS and histopathology provided the highest sensitivity for detection of malignancy (83%) at high specificity (99%) [4]. However, tissue acquisition is invasive and can be technically challenging, particularly in advanced strictures and areas that cannot be endoscopically reached. Therefore, liquid biopsy using bile‐derived cell‐free DNA (cfDNA) has emerged as a promising complementary approach [5]. Because bile is in direct contact with the biliary epithelium, it contains higher concentrations of tumour‐derived DNA than plasma [6]. In addition, while biopsies strictly sample a specific lesion, analysis of bile offers the availability to collectively sample a larger region, theoretically the entire biliary tree proximal to the sampling location. This may be of particular interest in patients with multiple strictures, for example in primary sclerosing cholangitis (PSC). Several studies have demonstrated a high concordance between genomic alterations detected in bile cfDNA and matched tumour tissue, suggesting that bile may serve as a reliable source for molecular profiling [7, 8, 9]. Subsequently, a next‐generation sequencing (NGS) panel, Bilemut, was developed for the analysis of bile‐derived cell‐free DNA (cfDNA) obtained during ERCP [5]. The assay demonstrated a sensitivity of 96.4% and a specificity of 69.2% for the detection of malignancy; however, its clinical utility was limited by a relatively high false‐positive rate of 15% [5]. Despite these advantages and the minimally invasive approach of liquid biopsy, bile cfDNA still cannot replace tissue biopsy. Molecular alterations alone do not provide histological confirmation of malignancy, and standardized protocols for sample processing, sequencing and interpretation are still lacking [6, 10]. Patients with PSC constitute a particularly relevant subgroup, as they are at increased risk of cholangiocarcinoma (CCC). Still, reliable risk stratification tools remain elusive, and timely diagnosis is often challenging, contributing to a poor prognosis with a median survival of only 5–7 months after diagnosis [11]. Remarkably, BiliSeq had a 83% sensitivity in patients with PSC compared with an 8% sensitivity of histopathological evaluation alone [4]. Furthermore, another study showed that NGS‐based mutation profiling of brush samples can substantially enhance cytological assessment in PSC, with high sensitivity (75%) and specificity (85%) for the identification of malignant alterations [12]. Beyond tissue‐based NGS approaches, a subsequent study building on the Bilemut panel, which introduced NGS analysis of bile cfDNA, demonstrated an association between bile cfDNA mutational profiles and malignancy risk in patients with PSC [13]. Consequently, NGS‐based testing represents a promising diagnostic approach, particularly for this high‐risk patient population.

Despite these encouraging findings, independent validation in real‐world clinical settings is required. Consequently, additional data are needed to assess the robustness, generalizability and clinical utility of this approach in routine practice [14] before the implementation of NGS in standard clinical diagnostics.

## 2 Patients and Methods

We conducted a single‐centre study to evaluate the performance of NGS analysis of CCC‐associated mutations in biopsies obtained by endoscopic retrograde cholangiography (ERC) in routine clinical care at the University Medical Center Dresden, Germany. Biopsies were obtained from biliary strictures of unknown aetiology by ERC, followed by NGS and analysis of the gene panel, similar to the approach reported by Singhi et al. [4]. All samples were derived from biliary forceps biopsies. We compared the diagnostic performance of NGS with that of serum CA19‐9, pathological evaluation and cross‐sectional imaging (magnetic resonance imaging or contrast enhanced computed tomography). In line with the cutoffs reported by Singhi et al. [4], CA19‐9 levels ≥ 44 U/mL (and > 129 U/mL in PSC) were considered elevated and therefore suspicious for malignancy. Patients were followed up until either confirmation of malignancy by histopathological evaluation or radiologic follow‐up without signs of malignant progression for at least 6 months. Singhi et al. [4] reported sensitivity for the detection of malignancy of 48% for histopathological evaluation alone vs. 83% for combined histopathological evaluation and BiliSeq. Assuming a malignancy rate of 50%, we calculated that a sample size of 32 patients would provide 90% power to detect a 30% difference in the sensitivity of malignancy detection between histopathological evaluation alone and combined histopathological evaluation and NGS. Sensitivity was compared between tests by the McNemar test.

### 2.1 NGS Methodology

Genomic DNA was isolated from macrodissected formalin‐fixed, paraffin‐embedded (FFPE) tissue sections using the QIAamp DNA Micro Kit (Qiagen) according to the manufacturer's protocol. Target enrichment was performed using the QIAseq Targeted DNA Panel protocol (Qiagen), followed by amplification of the coding regions. Libraries were sequenced in paired‐end mode on the Illumina platform, Illumina MiSeq and Illumina MiSeqDx.

The median sequencing depth across all samples was 2460×. Sequence reads were aligned to the human reference genome hg19 (GRCh37, February 2009 release), and bioinformatic analysis was performed using CLC Biomedical Workbench (Qiagen).

For annotation of variants, dbSNP, COSMIC, ClinVar, CIViC, Jax CKB and OncoKB were used. Although no formal limit of detection (LOD) validation study was performed, variants were called using a predefined VAF threshold of 5% for non‐hotspot regions. For selected hotspot variants, the analytical sensitivity was extended to detect variants with VAFs as low as 1%. Samples were analysed using the following panels:

Targetlist nNGM Panel (Version 1.0) *# Gene (ENSEMBL Transkript ID, RefSeq Transkript ID, sequenced Region): ALK (ENST00000389048, NM_004304, Exon 22–25), BRAF (ENST00000288602, NM_004333, Exon 11, 15), CTNNB1 (ENST00000349496, NM_001904, Exon 39), EGFR (ENST00000275493, NM_005228, Exon 18–21), ERBB2 (ENST00000269571, NM_004448, Exon 8, 19, 20), FGFR1 (ENSP00000400162, NM_023110, Exon 4–7, 10, 12, 13–15), FGFR2 (ENST00000358487, NM_000141, Exon 6–15, 18), FGFR3 (ENST00000440486, NM_000142, Exon 3, 6, 7, 9, 10, 12, 14, 16, 18), FGFR4 (ENST00000292408, NM_213647, Exon 3, 6, 9, 12, 13, 15, 16), IDH1 (ENST00000345146, NM_005896, Exon 4), IDH2 (ENST00000330062, NM_002168, Exon 4), KRAS (ENST00000256078, NM_033360, Exon 2–4), MAP2K1 (ENST00000307102, NM_002755, Exon 2, 3), MET (ENST00000318493, NM_001127500, Intron 13, 14 + Exon 14, 16–19), NRAS (ENST00000369535, NM_002524, Exon 2–4), PIK3CA (ENST00000263967, NM_006206, Exon 10, 21), PTEN (ENST00000371953, NM_000314, Exon 1–8), ROS1 (ENST00000368508, NM_002944, Exon 34–41), TP53 (ENST00000269305, NM_000546, Exon 4–8).

Targetlist ‘Bili Panel’ #: AKT1 (NM_005163, ENST00000555528.5 all Exons), CDKN2A (NM_000077, ENST00000304494.9 all Exons), GNAS (NM_080425, ENST00000371100 Exon 8), IDH2 (NM_002168; ENST00000330062 Exon 4), SMAD4 (NM_005359, ENST00000342988.8 all Exons), PIK3CA (NM_006218, ENST00000263967 Exon 8).

## 3 Results

**FIGURE 1:**
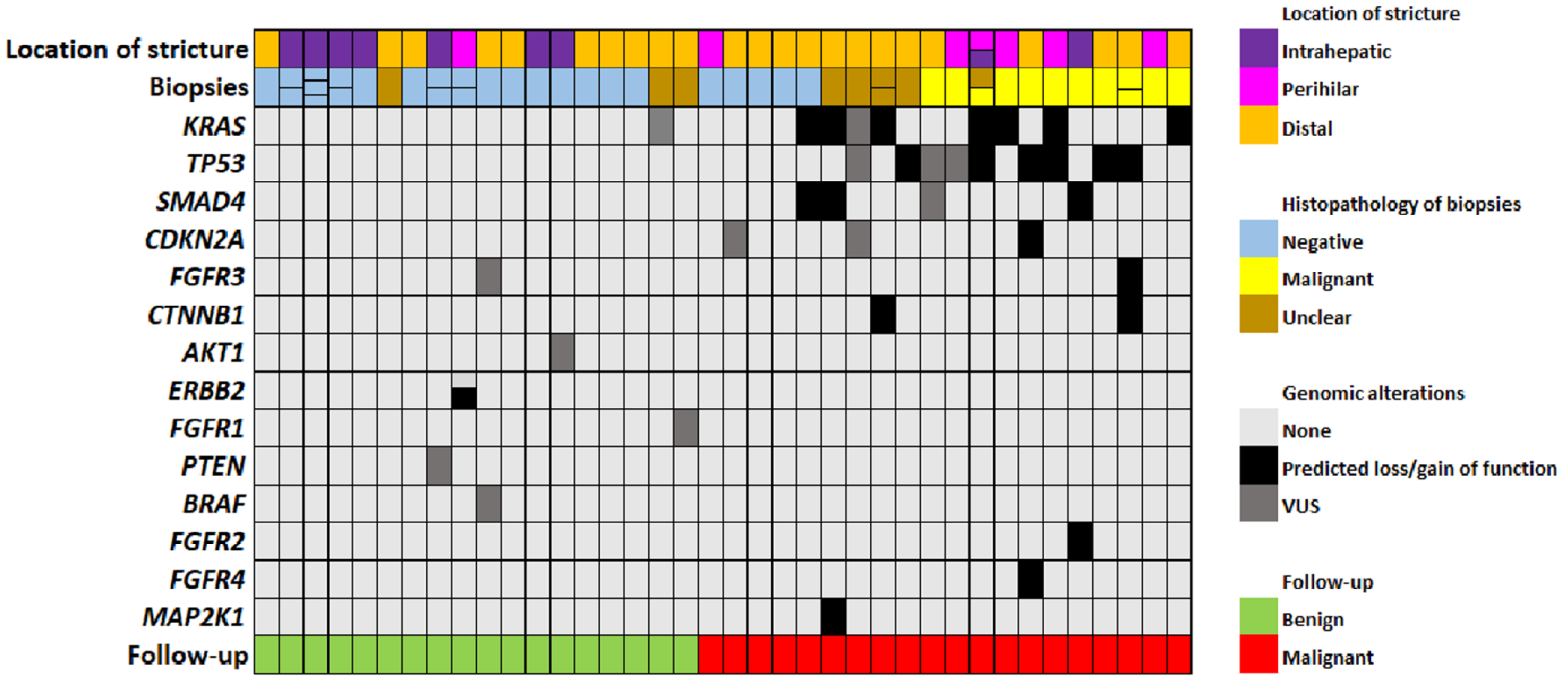
Visualization of stricture location and results of NGS analysis, histopathology and follow‐up. Each column represents one patient. Patients with multiple biopsies are shown as split cells.

We obtained biliary biopsies from 38 patients in 47 ERCs between 04/2019 and 05/2023. The majority of patients were male (*n* = 29, 76%), median age was 62 years (IQR 17.5 years), and four patients had primary sclerosing cholangitis. Median follow‐up was 6.3 months (IQR 10.5 months). Strictures were mostly located in the common bile duct (60%), while 24% were intrahepatic and 16% perihilar. Malignancy was ultimately detected in 20 of 38 patients (53%) and in 23 of 47 biliary specimens (49%). NGS analysis showed predicted gain or loss of function variants and/or VUS in 26 biopsies obtained from 22 patients (Figure 1). Eight patients underwent repeated ERC with biopsy sampling, with 7 of 8 patients showing identical NGS results in repeated sampling (one patient showed an *ERBB2* mutation only in a follow‐up ERC but not initially). Genetic alterations were most commonly observed in *KRAS* (24%), *TP53* (24%) and *SMAD4* (11%) and genes affected differed between patients with and without malignancy (Figure 1). The sensitivity and specificity of NGS for malignancy, determined at the level of individual biopsies, were 65% and 96%, respectively (Table 1), and thus remarkably similar to that described by in the publication by Singhi et al. (73% sensitivity, 100% specificity) [4]. In comparison, pathological evaluation (performed in all patients), elevated serum CA19‐9 (performed in 33 of 47 samples, 70%) and radiological imaging (performed in 37 of 47 samples, 79%) showed sensitivities of 52%, 65% and 40%, and specificities of 100%, 77% and 88%, respectively (Table 1). The combination of NGS with histopathological evaluation increased the sensitivity to 78% (*p* = 0.031) without negative effect on specificity (96%). In contrast, the combination of NGS with CA19‐9 or imaging increased sensitivity at the cost of reduced specificity. Inclusion of variants of unknown significance (VUS) in the 28 genes similarly increased sensitivity at the cost of specificity (Table 1). Molecular alterations affecting genes with potential therapeutic relevance [15], including KRAS, ERBB2, FGFR1, FGFR2 and MAP2K were detected.

**TABLE 1:** Diagnostic performance of NGS, histopathology, imaging and CA19‐9 for differentiation of biliary strictures expressed per biopsy.

	Sensitivity	Specificity	PPV	NPV
Individual analysis of parameters				
NGS analysis without VUS (with VUS); *n* = 47	65% (83%)	96% (71%)	94% (73%)	74% (81%)
Histopathology of biopsies; *n* = 47	52%	100%	100%	69%
Imaging; *n* = 37	40%	88%	80%	56%
CA 19–9; *n* = 33	65%	77%	81%	59%
Combined analysis of parameters				
NGS without VUS (with VUS) + Histopathology; *n* = 47	78% (87%)	96% (71%)	95% (74%)	82% (85%)
NGS without VUS (with VUS) + Imaging; *n* = 37	80% (90%)	82% (59%)	84% (72%)	78% (83%)
NGS without VUS (with VUS) + CA 19–9; *n* = 33	90% (95%)	77% (69%)	86% (83%)	83% (90%)
NGS without VUS (with VUS) + Histopathology + CA 19–9; *n* = 33	90% (95%)	77% (69%)	86% (83%)	83% (90%)

*Note:* Analysis of individual parameters is shown in the upper part, combined analysis in the lower part. Values in brackets show diagnostic performance if VUS are considered pathogenic.

Abbreviations: CA 19–9, carbohydrate antigen 19–9; NPV, negative predictive value; PPV, positive predictive value.

## 4 Discussion

Our results document that NGS analysis of CCC‐associated variants in biliary biopsies increases sensitivity for the detection of malignancy compared to histopathological analysis, without compromising specificity. The combination of histopathology and NGS analysis further increases sensitivity compared to NGS analysis or histopathology alone, again at comparable specificity. We identified molecular alterations affecting genes with potential therapeutic relevance [15]. Although the specific molecular alterations were not further characterized with respect to their therapeutic actionability, the identification of alterations in genes such as KRAS, ERBB2, FGFR1, FGFR2 and MAP2K highlights the potential of NGS to identify patients who may benefit from comprehensive molecular profiling and subsequent precision oncology approaches [15, 16].

A limitation of this study is the relatively small cohort size, which may affect the precision of diagnostic accuracy estimates and limits subgroup analyses, particularly in PSC patients. Although several studies have evaluated the use of next‐generation sequencing (NGS) for indeterminate biliary strictures in recent years, most have been limited by retrospective or single‐centre designs and relatively small patient cohorts [4, 5, 14, 15]. A recently published large prospective multicentre study has now provided robust evidence supporting the clinical value of NGS in this setting [15]. While several studies evaluated NGS in biliary strictures, reported diagnostic performance varies widely across cohorts, likely reflecting differences in patient selection, prevalence of malignancy, PSC representation and sampling as well as analysis strategies [15]. Consequently, well‐characterized independent cohorts remain important for validating previous findings and refining their applicability to specific clinical settings [14]. An additional limitation of this study is the relatively short follow‐up period used to exclude malignancy in patients without histological confirmation. Although a minimum follow‐up of 6 months has been applied in previous studies of indeterminate biliary strictures [14], longer surveillance may be required to detect slowly progressive malignancies, particularly indolent cholangiocarcinoma or PSC‐associated cancer. Consequently, the possibility of misclassification of benign cases cannot be entirely excluded, which may have influenced the reported diagnostic performance. Altogether, our data are in line with previous findings and support the inclusion of NGS analysis in the diagnostic workup of biliary strictures of unknown aetiology.

## Author Contributions

A.G. and S.Z. designed and coordinated the study and wrote the manuscript with input by all coauthors. R.S., S.B., J.Ha. and S.Z. included patients and collected specimens. D.A. performed the histopathological evaluation and NGS. Data analysis was done by A.G., J.Hu. and S.Z. The original draft was written by A.G. and S.Z. Review and editing were done by J.Ha., J.Hu., S.B., D.A. and R.S. All authors discussed the findings discovered after analysis and approved the final manuscript.

## Funding

The authors have nothing to report.

## Ethics Statement

The displayed data has been collected during clinical routine treatment. The analysis of this data has been approved by the local ethics committees of the Technical University Dresden (BO‐EK‐396092023). All data were processed and analysed in an anonymized manner.

## Conflicts of Interest

The authors declare no conflicts of interest.

## Data Availability

The data that support the findings of this study are available from the corresponding author upon reasonable request.
